# Prevalence of gaming addiction and its impact on sleep quality: A cross-sectional study from Pakistan

**DOI:** 10.1016/j.amsu.2022.103641

**Published:** 2022-04-20

**Authors:** Musharaf Zaman, Muhammad Saad Babar, Maryam Babar, Faheem Sabir, Farzana Ashraf, Muhammad Junaid Tahir, Irfan Ullah, Mark D. Griffiths, Chung-Ying Lin, Amir H. Pakpour

**Affiliations:** a Ameer-ud-Din Medical College, Affiliated with University of Health Sciences, Lahore, Pakistan; b Lahore General Hospital, Lahore, Pakistan; c Lahore Medical and Dental College, Lahore, Pakistan; d Department of Humanities, COMSATS University, Lahore, Pakistan; e Kabir Medical College, Gandhara University, Peshawar, Pakistan; f International Gaming Research Unit, Psychology Department, Trent University, Nottingham, United Kingdom; g Institute of Allied Health Sciences, College of Medicine, National Cheng Kung University, Tainan, Taiwan; h School of Health and Welfare, Jönköping University, Sweden

**Keywords:** Gaming, Gaming addiction, Sleep quality, Prevalence, Pakistani gaming

## Abstract

**Background:**

Gaming addiction has become a topic of increasing research interest worldwide but little research has been carried out in Pakistan.

**Aims:**

The present study assessed the prevalence of gaming addiction among a Pakistani sample of adults in the general population. It also explored the effects of online gaming addiction upon sleep quality.

**Method:**

A cross-sectional survey was carried out during a national lockdown due to the COVID-19 pandemic in Pakistan. Using a convenience sampling technique, an online survey comprising demographic information, the Game Addiction Scale (GAS), and the Pittsburgh Sleep Quality Index (PSQI) was completed by 618 participants (67.5% male) aged 18–56 years (*M* = 24.53 years, *SD* = ±5.016).

**Results:**

Out of 618 participants, 57.0% (n=352) played online games. Among gamers, 12.5% (n = 44) were classed as addicted to the gaming based on GAS scores. Compared to those not addicted to gaming, participants with gaming addiction had significantly poorer subjective sleep quality, higher sleep disturbance, lesser sleep duration, and higher daytime dysfunction. Gaming addiction was also more prevalent among males compared to females.

**Conclusion:**

Gaming addiction among the Pakistani general population is significantly associated with poor sleep quality. This problem needs to be addressed at both individual and societal levels to avoid adverse long-term health impacts.

## Highlights

• Problematic gaming behavior has been identified as one of the emerging public health issues, especially among adolescents and young adults.
• Gaming addiction in the Pakistani population affected a significant minority (based on GAS scores) and was associated with poor sleep quality.
• Gaming addiction was found to be more prevalent among males compared to females.
• Psychoeducation on the knowledge of gaming for general population of Pakistan may be used to be a preventive method to improve their sleep.

## 1 Introduction

In the modern era, individuals spend a lot of time on their smartphones, social media, and playing online games on different devices [1]. While the vast majority of individuals find these activities of great benefit, a small proportion may experience problems. More specifically, problematic gaming behavior has been identified as one of the emerging public health issues, especially among adolescents and young adults [2]. Indeed, a growing body of literature on the topic of problematic gaming or internet addiction can be observed in the past decade [3]. In this regard, it is important for healthcare providers to more deeply understand the phenomenon related to problematic gaming, including its prevalence in every single country and impacts on health.

Problematic videogame playing can have several effects on an individual's physical and mental health and can impact upon an individual's daily activities and changes their behavior [1]. The American Psychiatric Association has now included internet gaming disorder (IGD) as a tentative disorder that highlights the need for further exploration by researchers in this area [4] Neurobiological research on gaming has shown that when a gamer ‘levels up’ and acquires more expensive weapons, more dopamine is released in some brain regions, which reinforces the behavior and helps explain why the gaming may be addictive for some [5,6]. Individuals who are addicted to games lose interest in their real life and some simply focus on how they can achieve more in the game [7]. Such problematic behavior can lead to psychosocial problems, sleep disturbances, relationship problems, inability to focus on minor tasks, and other mental health pathologies [8,9]. It has also been associated with stress, mood modification, and poor academic performance [10].

Gaming addiction and other types of problematic internet use also appear to have a significant influence on the sleep-wake cycle, leading to insomnia and other sleep disturbances [11]. Sleep quality can be assessed in a number of domains such as how easy it is for an individual can go to sleep, the number of sleeping hours, and how refreshed individuals feel upon awakening [5]. Previous studies have shown that increased time spent on online gaming and internet use leads to several sleep disturbances including low sleep quality, sleep delays, irregular sleeping patterns, and excessive daytime sleepiness [[12], [13], [14]].

One explanation of the negative impact of problematic online gaming on sleeping habits may be due to high arousal which may interfere with body soothing processes necessary for sleep [15].Playing online games is also responsible for secretion of neurotransmitters in the reticular activating system of the brain. Gaming can also result in the release of stress hormones, especially when there is a difficult situation in the game that needs the gamers paying high attention and focus [5]. The literature also shows that constantly playing online videogames can negatively affect sleep quality and is associated with shorter REM sleep [9]. Moreover, online games allow individuals from different geographical regions to team up and play a game together. Therefore, gamers from a different time zone can keep themselves awake or get up in the middle of the night to play online games, have a low sleep quality, and feel tired the rest of the day [9,13]. As a result, when individuals are addicted to gaming, they are easily to encounter different types of sleep problems.

Although the importance of understanding the impacts of problematic gaming is well acknowledged in the literature, few studies have been conducted in Pakistan to explore the prevalence of gaming addiction [3]. One Pakistani study explored the association between time spent playing online games and problematic gaming and found that spending more than 41 h per week playing online games was associated with internet gaming disorder (IGD) [16]. Another study conducted among Pakistani adolescents showed that gaming addiction was associated with anger expression, narcissistic personality, and had a negative relationship with social interactions [7]. The same study also reported that gamers with addiction problems had social circles with other gamers, and that they used games as a source of communication with them [7]. A more recent study on Pakistani university students revealed that 50.8% of the students having the problems of gaming addiction or were at risk of developing gaming addiction [17]. Aside from the aforementioned studies, to the best knowledge of the present authors, no other evidence on problematic gaming (or gaming addiction) has been reported using a general population in Pakistan. Therefore, in order to extend the empirical base of gaming research in Pakistan, the present study explored the prevalence of gaming addiction in the Pakistani population and how it affects sleep quality.

## 2 Method

### 2.1 Participants and study design

A cross-sectional survey study was conducted between June and July 2020 and collected data from the Pakistani general population using *Google Forms*. Power analysis estimated a minimum of 600 participants to reliably assess the effects of online gaming on sleep habits and sleep quality with a 4% margin of error and with a 95% confidence level using *Raosoft.* The study collected data from 618 participants (67.5% male; 56.3% aged 23–27 years of age). For the present study, the inclusion criteria were (i) being Pakistani and (ii) being adult (aged 18 years or older). The study was approved by COMSATS University Islamabad, Lahore Campus ethics committee (Ref no: 2577) and was carried out in accordance with human research ethics outlined in the Helsinki Declaration, 1975. The present study was reported in line with the STROCSS criteria [18].

### 2.2 Measures

The online cross-sectional survey comprised three sections: questions concerning demographic information, a scale to assess gaming addiction, and a scale to assess sleep quality.

*Demographic information*: This section asked about participants’ age (in years), gender (male or female), education (no formal education, primary, secondary, higher secondary, graduate, post-graduate, and other), marital status (single, married, divorced, widowed, separated), occupation, residence (urban, rural, semi-urban), and family monthly income. Two additional questions concerned the playing of online games (yes or no), and whether the number of hours spent gaming had increased, decreased or stayed the same during lockdown.

*Assessment of gaming addiction:* Although there are many instruments that have been developed to assess problematic gaming [[19], [20], [21]], the Gaming Addiction Scale (GAS) developed by Lemmens et al. [22] was used in the present study. The GAS comprises seven items each assessing a different dimension of gaming addiction (i.e., salience, tolerance, mood modification, relapse, withdrawal, conflict, and problems) and each item is rated on a five-point Likert scale from 1 (*never*) to 5 (*very often*). A higher score on the GAS indicates more problematic use of online gaming. The GAS classifies gamers into four types: (i) *addicted gamers* (scoring 3 or more on the four final items [relapse, withdrawal, conflict, and problems]), (ii) *problem gamers* (scoring 3 or more on two or three of the four final items), (iii) *engaged gamers* (scoring 3 or more on the first three items [salience, tolerance, and mood modification] but who did not score three or above on more than one of the final four items, and (iv) *normal gamers* (those not classified as addicted, problem, or engaged gamers) [22]. The internal consistency (Cronbach's alpha) for the GAS in the present study was very good (0.803).

*Assessment of sleep quality:* The Pittsburgh Sleep Quality Index (PSQI) was used to assess sleep quality. The PSQI comprises 19 items assessing a wide variety of factors related to sleep quality in the past month [23], including sleep duration, sleep latency, sleep frequency, and severity of specific sleep-related problems. Each item on the PSQI assesses sleep quality on a scale from 0 to 3. The seven item scores are added together for a global score. Total scores range between 0 and 21, and high scores indicate greater sleep problems. The PSQI scores can also be used to classify individuals as having poor sleep and good sleep. A total score of 6 or more is deemed to indicate poor sleep. The internal consistency (Cronbach's alpha) for the PQSI in the present study was good (0.70).

### 2.3 Study procedure

**Fig. 1:**
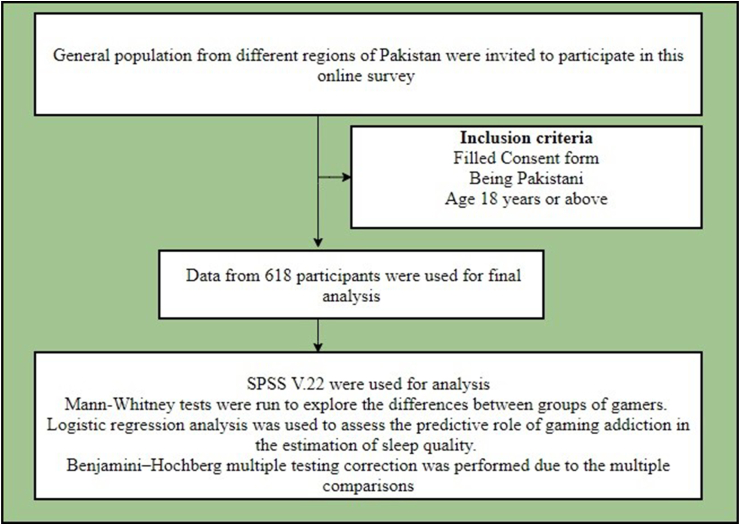
Flowchart of the study.

The sample was recruited using a convenient sampling technique. Data were collected by sharing the survey link on different popular Pakistani social media sites (e.g., *WhatsApp, Facebook, Instagram*). The detailed flowchart of this study is shown in Fig. 1. The study was approved by the research team's university ethics committee. All the participants provided informed consent to take part in the study. The survey was divided into three sections comprising demographic data (i.e., age, gender, profession, education, etc.), the Gaming Addiction Scale, and the Sleep Quality Index.

### 2.4 Statistical analysis

The data were analyzed using SPSS (version 22). Initially, outliers were identified and missing values analysis was performed to ensure normal distribution of data. Following that, inferential statistics were also applied to test study assumptions. Descriptive analysis was used to assess study variables including means, standard deviations, percentages, and frequencies. Chi-square tests of association were applied to examine the associations between study variables. Mann Whitney tests were run to explore the differences between groups of gamers. Logistic regression analysis was used to assess the predictive role of gaming addiction in the estimation of sleep quality. A *p*-value <.05 was used to differentiate significant and non-significant differences. Benjamini–Hochberg multiple testing correction was performed due to the multiple comparisons.

## 3 Results

Out of 618 participants, 266 did not play online games (43.0%), while 352 did play online games (57.0%). Among online gamers, most (67.3%) reported that gaming hours had increased (from slightly to almost double) during the lockdown due to the COVID-19 pandemic (67.3%). The other descriptions are presented in Table 1. The sentence should start from "The analysis also found that among those who were gamers, 44 were classed as addicted gamers, 44 were classed as addicted gamers (12.5%) using the GAS, 156 of the gamers were problem gamers (44.3%), and 20 were engaged gamers (5.7%), and 132 were normal gamers (37.5%). Among the subsample of gamers, there was significant relationship between gaming and subjective sleep quality (*p* < .001), sleep disturbance (*p* = .001), daytime dysfunction (*p* < .001) and sleep duration (*p* = .002) in normal gamers and the engaged gamers, problem gamers and addicted gamers (Table 3). Subjective sleep quality (*p* = .610), sleep latency (*p* = .397), sleep duration (*p* = .196), sleep disturbance (*p* = .705) and use of sleep medication (*p* = .269) had no significant relationship among online gamers (n = 363) and non-gamers (n = 279). Daytime dysfunction (*p* = .009) had a d significant relationship among online gamers (n = 363) and non-gamers (n = 266) (see Table 2).

**Table 1:** Association of sociodemographic characteristics of participant with study variables.

Characteristics	Gender			Chisquare		Logistic Regression (gaming addiction)		
	Male	Female	Total	Benjamini–Hochberg corrected *p*-values	*ꭓ*^2^	*p*	*OR*	*CI 95% (UL-LL)*
Age (years)	*F (%)*	*F (%)*	*F (%)*	0.625	1.749	.0001		
18-22	130 (69.5)	57 (30.5)	187 (30.3)			.003	3.167	1.495 to 6.708
23-27	236 (67.8)	112 (32.2)	348 (56.3)			.710	1.147	.556 to 2.36
>27 ^(Ref)^	51 (61.4)	32 (38.6)	83 (13.4)					
Resident				0.132	5.442	.095		
Rural	46 (78.0)	13 (22.0)	59(9.5)			.031	3.031	1.106 to 8.30
Urban	297 (65.0)	160 (35.0)	457(73.9)			.320	1.400	.721 to 2.71
Semi urban ^(Ref)^	74 (72.5)	28 (27.5)	102(16.5)					
Marital status				0.003	15.606	.256		
Single	362 (70.8)	149 (29.2)	511(82.7)			.952	.944	.148 to 6.04
Married	49 (50.5)	48 (49.5)	97(15.7)			.394	.432	.062 to 2.98
Divorced ^(Ref)^	06 (60.0)	04 (40.0)	10(1.6)					
Family Income				0.756	1.187	.028		
<30000	54 (64.3)	30 (35.7)	84(13.6)			.042	.397	.163 to .96
30000-60000	102 (70.3)	43 (29.7)	145(23.5)			.149	1.568	.851 to2.88
60001-90000	127 (68.3)	59 (31.7)	186(30.1)			.714	.894	.493 to 1.62
>90000 ^(Ref)^	134 (66.0)	69 (34.0)	203(32.8)					
						**Logistic Regression (sleep quality)**		
Play online game				0.003	26.147			
No	150 (56.4)	116 (43.6)	266(43.0)			.026	1.440	1.044 to 1.987
Yes ^(Ref)^	267 (75.9)	85 (24.1)	352(57.0)					
Gaming hours during lockdown				0.756	4.379	.049		
Decrease more than half	13 (92.9)	01 (7.3)	14(2.34)			.448	1.666	.446 to 6.21
Decrease to half	06 (75.0)	02 (25.0)	8(2.3)			.233	.384	.079 to 1.85
Decreased slightly	08 (61.5)	05 (38.5)	13(3.7)			.171	.416	.118 to 1.46
Remained same	58 (72.5)	22 (27.5)	80(22.7)			.122	.560	.268 to 1.16
Increased slightly	86 (76.8)	26 (23.2)	112(31.8)			.004	.362	.181 to .72
Increased to double	53 (77.9)	15 (22.1)	68(19.3)			.067	.497	.235 to 1.05
Increased more than double ^(Ref)^	43 (75.4)	14 (24.6)	57(16.2)					

**Characteristics**	**Gender**			**Chi Square**		**Logistic Regression**		
	**Male**	**Female**	**Total**	**Benjamini–Hochberg corrected *p*-Values**	***ꭓ***^**2**^	***p***	***OR***	***CI 95% (UL-LL)***
Game addiction category				.421	4.145	**.014**		
Normal Gamers	97	35	132(37.5)			.003	.317	.149 to .670
Engaged Gamers	12	8	20(5.7)			.158	.449	.147 to 1.36
Problem Gamers	123	33	156(44.3)			.119	.562	.272 to 1.16
Addicted Gamers ^(Ref)^	35	9	44(12.5)					
Type of Gamers				.421	0.646			
Pathological Gamers	170	50	220 (62.5)			.0001	.353	.223 to .560
Normal Gamers ^(Ref)^	97	35	132 (37.5)					
Gender								
Male	–	–	–	–	–	**.082**	.633	.378 to 1.060
Female ^(Ref)^	–	–	–	–	–			

Ref = reference group.

**Table 2:** Mean scoring of PSQI items in addicted and non-addicted participants (Mann Whitney test).

PSQI Items	Normal gamers	Other gamers^a^	Benjamini–Hochberg corrected -*p*values	*z*
Subjective sleep quality	0.727 ± 0.643	1.118 ± 0.868	0.003	−4.108
Sleep latency	1.204 ± 0.914	1.386 ± 0.932	0.109	−1.675
Sleep duration	0.652 ± 0.800	0.986 ± 0.977	0.003	−3.146
Sleep efficiency	0.561 ± 0.867	0.827 ± 1.101	0.050	−2.062
Sleep disturbance	1.121 ± 0.567	1.354 ± 0.613	0.002	−3.476
Use of sleep medication	0.219 ± 0.543	0.323 ± 0.777	0.576	−0.560
Daytime dysfunction	0.614 ± 0.638	1.054 ± 0.900	0.003	−4.507

a Engaged gamers, problem gamers and addicted gamers.

**Table 3:** Association between sleep parameters and playing online games (Chisquare test).

Sleep Parameters	Playing online game, N (%)			*ꭓ*^2^*(df)*	Benjamini–Hochberg corrected *p*-values
	No, N = 266	Yes, N = 352	Total, N = 618		
**Subjective sleep quality**				1.823 (3)	0.705
Very good	76 (12.3)	100 (16.2)	176 (28.5)		
Fairly good	147 (23.8)	183 (29.6)	330 (53.4)		
Fairly bad	27 (4.4)	48 (7.8)	75 (12.1)		
Very bad	16 (2.6)	21 (3.4)	37 (6.0)		
**Sleep latency**				2.966 (3)	0.556
0	47 (7.6)	68 (11.0)	115 (18.6)		
1	99 (16.0)	149 (24.1)	248 (40.1)		
2	78 (12.6)	90 (14.6)	168 (27.2)		
3	42 (6.8)	45 (7.3)	87 (14.1)		
**Sleep duration**				4.691 (3)	0.467
>7 h	112 (18.1)	151 (24.4)	273 (42.6)		
6–7 h	80 (12.9)	127 (20.6)	207 (33.5)		
5–6 h	44 (7.1)	46 (7.4)	90 (14.6)		
<5 h	30 (4.9)	28 (4.5)	58 (9.4)		
**Sleep efficiency**				4.142 (3)	0.467
>85%	140 (22.7)	212 (34.3)	352 (57.0)		
75–84%	53 (8.6)	58 (9.4)	111 (18.0)		
65–74%	47 (7.6)	48 (7.8)	95 (15.4)		
<65%	26 (4.2)	34 (5.5)	60 (9.7)		
**Sleep disturbance**				1.400 (3)	0.705
0	16 (2.6)	23 (3.7)	39 (6.3)		
1	168 (27.2)	219 (35.4)	387 (62.6)		
2	73 (11.8)	103(16.7)	176 (28.5)		
3	9 (1.5)	07 (1.1)	16 (2.6)		
**Use of sleep medication**				3.927 (3)	0.467
Not during the past month	229 (37.1)	290 (46.9)	519 (84.0)		
Less than once a month	26 (4.2)	36 (5.8)	62 (10.0)		
Once or twice a week	8 (1.3)	14 (2.3)	22 (3.6)		
Three or more time a week	3 (0.5)	12 (1.9)	15 (2.4)		
**Daytime dysfunction**				11.628 (3)	0.043
0	94 (15.2)	127 (20.6)	221 (35.8)		
1	104 (16.8)	155 (25.1)	259 (41.9)		
2	63 (10.2)	52 (8.4)	115 (18.6)		
3	5 (0.8)	18 (2.9)	23 (3.7)		

Binary logistic regression demonstrated significant predictive role of some of the demographics partially; monthly income (*p* < .05; OR = 0.397; CI = 0.163 to 0.96) in determining gaming addiction. Moreover, the analysis also estimated the predictive role of gaming addiction in determining sleep quality and found significant effects (*p* < .05, OR = 1.440; CI = 0.1.044 to 1.987). Other significant predictive associations were observed in relation to gaming addiction category (*p* < .01; OR = 0.317; CI = 0.149 to 0.670) and types of gamer (*p* < .001; OR = 0.353; CI = 0.223 to 0.560) (see Table 3).

## 4 Discussion

In the present study, 352 participants played online games (57.0%), and were classified into two categories (gamers and non-gamers). The prevalence of gaming addiction using the GAS was 7.1% with a further 25.2% being classed as problematic gamers. Moreover, the present study found that gaming addiction was a strong predictor for poor quality of sleep (OR = 1.440) after controlling for potential confounders.

As compared with the recent Pakistani survey by Zahra et al. [17] who reported that 50.8% of university students were classed as having a gaming addiction or were at risk of developing gaming addiction, the present study found a lower prevalence of problematic gaming. Several reasons may explain the difference: First, the studied populations were different. Prior evidence shows that adolescents and young adults, especially university students, are the population with the highest risk of developing internet addiction, including problematic gaming [24]. Therefore, university students who were surveyed by Zahra et al. [17] are likely to have more severe problems in gaming than the present study's population. Second, the COVID-19 pandemic allowed individuals to have substantially increased free time for gaming given that they were restricted from engaging in social activities. It is postulated that the craving for gaming may have decreased when there was too much time spent gaming. Indeed, a recent longitudinal study found that schoolchildren had decreased problematic gaming during the COVID-19 outbreak period when compared to their problematic gaming before the COVID-19 outbreak [25]. Third, the measures used to determine problematic gaming were different between the two studies. Given that different measures may have different features in their sensitivity and specificity, it is possible to have some differences when different measures were used.

Participants in the present study with gaming addiction had low sleep quality, less sleep duration, sleep disturbance, and daytime dysfunction. Similar findings have been found across countries. For example, a study conducted in Indonesia showed a decline in sleep quality due to gaming addiction [5]. Another study investigated in Hong Kong reported a significant association between gaming addiction and poor sleep quality [9]. Spending so much time on the screen causes fatigue in the peri-orbital area, causing eye closure difficulty and negatively affecting sleep [26]. There is an increase in stress hormones in the body when playing online games, making it more active [27]. Playing online games cause norepinephrine release in the cortex and dopamine in the midbrain. These neurotransmitters are part of the reticular activating system (RAS) and keeping gamers awake [5]. These biological mechanisms can therefore provide reasonable explanation to the present study's findings that problematic gaming was associated with poor sleep.

Previous studies have shown that addiction to online gaming is associated with several other adverse effects on psychological health including depression, anxiety, and sleep quality [23]. Problematic gaming is also associated with nervousness, tiredness, and exhaustion [23]. Unfortunately, gamers, especially those who are addicted to gaming may experience the release of dopamine which acts as a strong stimulus in spending more time playing online games [28]. In other words, playing online games causes dopamine release in the brain's reward center and may help explain how individuals become addicted to gaming [28]. Rajab et al. reported that online gaming addiction is directly related to stress. This can be bidirectional because individuals play videogames to cope with stress, but gaming itself can elevate individuals' arousal levels and subsequently prevent them from good sleep [29]. However, the associations and underlying mechanisms between sleep, psychosomatic health, and gaming addiction are unstudied and there is little empiricalevidence in Pakistan. Given that behaviors are usually different across countries (e.g., sleep duration is longer among British population than among Taiwanese population) [30], assessing the associations in each single country is therefore needed. In this regard, there is a need for further studies that explore the prevalence of gaming addiction in different age groups to examine various factors that influence not only sleep but also the psychosomatic health of individuals in Pakistan.

### 4.1 Limitations

In the present study, there are several limitations. Firstly, data were collected using convenience sampling and the recruited sample were likely to share similar demographic characteristics. Consequently, results cannot be generalized. Secondly, the study did not assess psychological distress and thereforedid not have evidence on the association between online gaming addiction with depression, anxiety, and stress. Thirdly, the study did not explore addiction to specific games and some videogames may be more addictive than others. Finally, the sample size was modest.

## 5 Conclusion

Gaming addiction in the Pakistani population affected a significant minority (based on GAS scores) and it was associated with poor sleep quality. With the aforementioned findings, healthcare providers in Pakistan should pay attention to gaming behaviors among Pakistani individuals who report poor sleep. More specifically, healthcare providers can check whether an individual complaining of poor sleep has an issue with problematic gaming. Consequently, tackling the problem of gaming addiction may subsequently improve the individual's sleep. Moreover, psychoeducation concerning the knowledge of gaming among the general population of Pakistan may be used as a a preventive method to improve their sleep.

## Ethical approval

Ethical approval was provided by the Ethics Committee at COMSATS University Islamabad, Lahore Campus, and adhered to the Helsinki Declaration.

## Ethics statement

All procedures performed in studies involving human participants were carried out accoding to the ethical standards of the Health Research Ethics Committee.

## Funding

None.

## Consent

We requested the participant's consent to publish this study for educational purposes.

## Registration of research studies

**Name of the registry**: This study was registered with the Ethics committee of COMSATS University Islamabad, Lahore Campus.

**Unique Identifying number or registration ID**: COMSATS University Islamabad (Ref no: 2577).

**Hyperlink to your specific registration (must be publicly accessible and will be checked):**

## Author contribution

IU and MJT conceived the idea; MZ, SB, MB, and FS collected the data; IU analyzed and interpreted the data; MZ, SB, MJT, FS, FA and MB did write up of the manuscript; and finally, FA, IU, MDG, CYL and AHP reviewed the manuscript for intellectual content critically. MDG edited the final manuscript. All authors contributed significantly to and edited all sections of the manuscript and have approved the final version.

## Data availability

Data will be made available on reasonable request to the corresponding authors.

## Provenance and peer review

Not commissioned, externally peer reviewed.

## Declaration of competing interest

The authors declare that they have no conflict of interest.
